# Engineering Tumor-Targeting Nanoparticles as Vehicles for Precision Nanomedicine

**DOI:** 10.20900/mo.20190021

**Published:** 2019-09-30

**Authors:** Amber Gonda, Nanxia Zhao, Jay V. Shah, Hannah R. Calvelli, Harini Kantamneni, Nicola L. Francis, Vidya Ganapathy

**Affiliations:** 1 Department of Biomedical Engineering, Rutgers, The State University of New Jersey, 599 Taylor Road, Piscataway, NJ 08854, USA; 2 Department of Chemical and Biochemical Engineering, Rutgers, The State University of New Jersey, 98 Brett Road, Piscataway, NJ 08854, USA; 3 Department of Molecular Biology and Biochemistry, Rutgers, The State University of New Jersey, 604 Allison Road, Piscataway, NJ 08854, USA

**Keywords:** nanoparticles, nano-bio interactions, nanotechnology, targeting, nanomedicine, cancer, theranostic

## Abstract

As a nascent and emerging field that holds great potential for precision oncology, nanotechnology has been envisioned to improve drug delivery and imaging capabilities through precise and efficient tumor targeting, safely sparing healthy normal tissue. In the clinic, nanoparticle formulations such as the first-generation Abraxane® in breast cancer, Doxil® for sarcoma, and Onivyde® for metastatic pancreatic cancer, have shown advancement in drug delivery while improving safety profiles. However, effective accumulation of nanoparticles at the tumor site is sub-optimal due to biological barriers that must be overcome. Nanoparticle delivery and retention can be altered through systematic design considerations in order to enhance passive accumulation or active targeting to the tumor site. In tumor niches where passive targeting is possible, modifications in the size and charge of nanoparticles play a role in their tissue accumulation. For niches in which active targeting is required, precision oncology research has identified targetable biomarkers, with which nanoparticle design can be altered through bioconjugation using antibodies, peptides, or small molecule agonists and antagonists. This review is structured to provide a better understanding of nanoparticle engineering design principles with emphasis on overcoming tumor-specific biological barriers.

## INTRODUCTION

Humanity’s earliest exploration of nanomaterials dates back to the 14th century B.C., when metallic nanoparticles, composed of gold and silver, were used to improve optical properties and visual aesthetics of glass artifacts [1]. However, it was not until the late 1950s, when physicist Richard Feynman proposed the method of manipulating and controlling individual atoms and molecules, that nanoscale engineering of materials was envisioned [2]. Nanomaterials possess a range of unique chemical and physical properties, which led to rapidly growing interest and opened doors for a wide range of applications, including flexible adaptation to industry sectors. Of note, some revolutionary industry products involving nanomaterials include high-end applications in aerospace construction, military designs such as biosensors and camouflage clothing, and medicine [3].

In the field of nanomedicine, nanoparticles (NPs) show great potential in oncology as drug carriers and enhanced imaging tools. NPs are defined as particles within the size range of 1–100 nm, where the presence of a large surface area allows for increased cellular interactions and multiple alterations of surface properties. NPs are currently at the forefront of research as delivery vehicles for medical imaging and therapy, especially in cancer therapy. However, the various advances made in understanding molecular cancer biology are minimally translated to a clinical stage due to the lack of ideal delivery mechanisms [4,5]. The inadequate translation is mainly due to the lack of an effective way to deliver therapeutic moieties or contrast agents to the target site with minimal side effects and negligible damage to the healthy tissue. An ideal delivery vehicle should be able to (1) increase selectivity of drug/contrast agent to target cells with improved pharmacodynamics and pharmacokinetics, [6,7] and (2) evade biological barriers and reach target sites efficiently [8]. The key advantages of NPs include their unique biological interactions based on their physical and chemical properties including charge, size, shape, and surface chemistry. Their high surface area to volume ratio also allows for loading therapeutics at a high concentration and dense display of targeting ligands, which can increase the localized effect by controlled release of the drug within targeted cells [9,10]. Additionally, integrating the diagnostic and therapeutic cargo in NPs holds promise for multimodal theranostic particles. Among the key attributes of nanoparticles that can be manipulated for prolonged circulation and improved delivery to the lesion are their size [11,12], surface properties [13], and presence of active targeting moieties [14].

A number of disease targeting therapeutics, as vehicles for precision nanomedicine, have been approved by the US Food and Drug Administration (FDA). One of the most successful has been that of the monoclonal antibody, which has over 60 formulations in clinical trials or in clinical use for a variety of pathologies, including cancer [15]. The number of FDA-approved nanoparticles in clinical use in oncology is much smaller and comprises mostly liposome-based formulations (Table 1) with the exception of Abraxane®. These include liposome-encapsulated doxorubicin, first approved for the treatment of HIV-related Kaposi’s sarcoma in 1995 (Doxil®), and later for the treatment of ovarian and breast cancer in 1999 (Caelyx®) [16]. The liposomal formulation of this anticancer drug not only provides a longer half-life and enhanced tumor deposition, but also lowers incidence of cardiotoxicity, myelosuppression, alopecia, and nausea [7,17]. In this review, we summarize the design criteria that guide the use of nanoparticles in cancer medicine in relation to the respective tumor niches/biological barriers. We will first elucidate the biological barriers in tumors from an organ level to the cellular level. Following this, we will describe the design conditions that will help overcome these barriers and discuss some of the nano-bio interactions.

## BIOLOGICAL BARRIERS FOR NANOPARTICLE DELIVERY TO TUMORS

**Figure 1.**
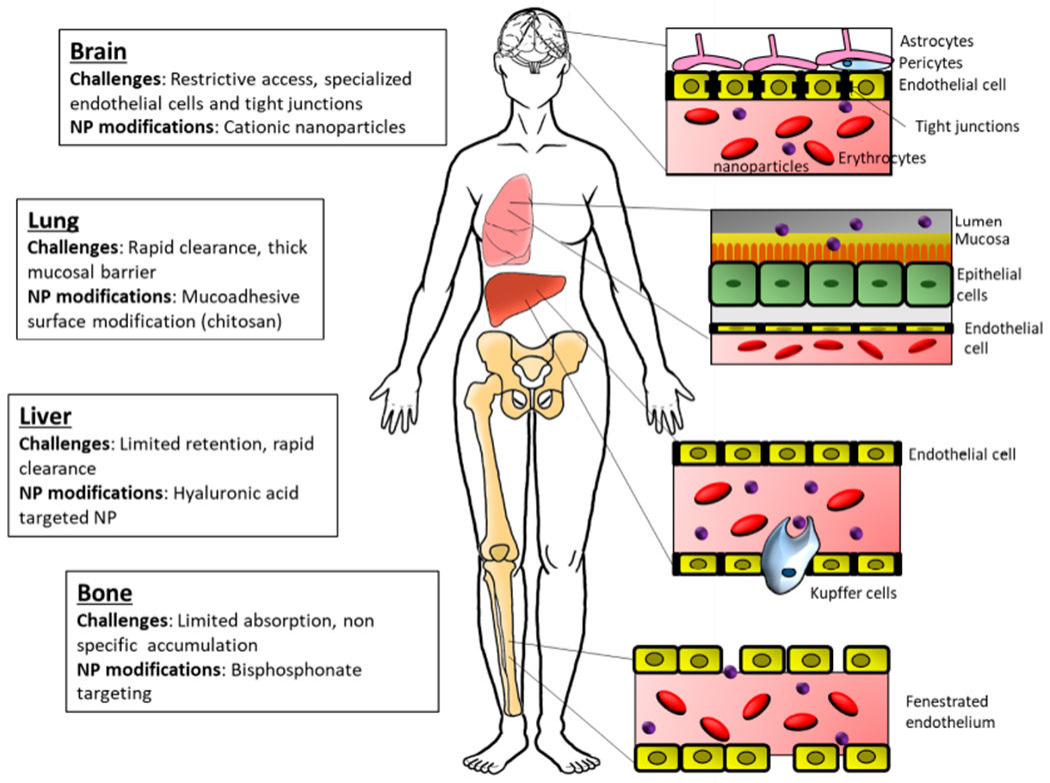
Biological barriers for nanoparticle delivery. The schematic highlights the barriers to nanoparticle delivery at common organs of tumor development and metastatic progression.

Development and optimization of nanotechnology in oncology requires a clear understanding of the biological barriers that facilitate or impede NP distribution and delivery. The anatomy and physiology of the tumor and the body present formidable biological barriers that protect the body from foreign material. As such, NP characteristics must be specifically tailored to address and overcome these obstacles in order to improve precise delivery of drugs and facilitate accurate diagnostic imaging. Biological barriers are present on a systemic, organ, and cellular level, creating a unique environment for each tumor type (Figure 1).

### Systemic Barriers

#### Biodistribution

One of the most challenging systemic obstacles facing the successful delivery of NPs is the biodistribution and clearance regulated by interdependent systems. Delivery of foreign substances to the body is impeded by structural and chemical processes which protect from exposure to harmful substances. Materials ingested encounter acidic environments, immune surveillance, and protective mucosal linings [19,20]. In the case of lung tumors, due to first pass pulmonary uptake, inhalation or intravenous administration are optimal with particles >100 nm [21]. The circulatory system likewise provides both size-restrictive properties as well as constant immune surveillance. The endothelial and basal membranes which are dependent on anatomical location [22] vary in pore size and can influence the selective localization of NPs (Figure 1). For instance, the blood vessels within the bone space consist of a discontinuous basal membrane and large gaps between the endothelial cells facilitating higher accumulation of nanoparticles. The lungs and endocrine glands, like the adrenals, however, have a continuous basal membrane with slightly fenestrated endothelial cells, resulting in lower accumulation of similar sized particles. The cumulative effect of endothelial pore size dictates the localization of NPs based on size. Additionally, the immune cells within the circulatory system and surrounding tissues, primarily the macrophages of the reticuloendothelial system (RES), present an active barrier to NP delivery via their rapid removal from circulation [23].

Tumor vasculature presents unique properties which affect the distribution and delivery of NPs. In the development of a tumor, angiogenesis is a dynamic process which facilitates continual cell proliferation and tumor growth by extending the availability of oxygen and other nutrients. Signaling molecules released by surrounding cells are essential in this process and include such proteins as vascular endothelial growth factor (VEGF-A) [24]. Over-secretion of VEGF drives rapid angiogenesis, the uncontrolled and rapid nature of which leads to the development of leaky vessels with increased permeability [25,26]. The “leaky” vasculature provides an opportunity for higher accumulation of NPs in the tumor, which has been termed the enhanced permeability and retention (EPR) effect [27]. In a recent study, Natfji *et al.* found that the highest accumulation of NPs in tumors via EPR occurred in pancreatic cancer, followed in order by colorectal cancer, breast cancer, gastrointestinal cancer, brain cancer, and ovarian cancer [28]. A recent analysis by Wilhelm *et al.*, also evaluated the effect of EPR-mediated accumulation of NPs and determined skin, pancreas, brain, and liver tumors to be the most common tumors with the highest accumulation of NPs [29]. However, in spite of this, fewer than 0.7% of nanoparticles reach the tumor site [29]. One of the factors that could improve nanoparticle delivery in tumors is the change in vasculature post-radiation therapy [30]. Radiation therapy has shown an increase in nanoparticle accumulation. Werner *et al.* [31], in their study in 2013, were able to show increased accumulation of liposomal paclitaxel for improved radiosensitivity in lung cancer. In a similar study involving gastric cancer, Cui *et al.* [32] were able to show enhanced effect of radiation therapy with the use of docetaxel nanoparticles. Ionizing radiation has been shown to increase tumor accumulation of nanoparticles by Giustini *et al.* through modifications in the tumor microenvironment via reduced interstitial fluid pressure and enhanced vascular permeability [33]. Despite this unique pattern of distribution, NP delivery to tumors remains low, indicating that the EPR effect is not sufficient on its own to ensure NP accumulation and activity [5,29]. One novel approach to overcoming the dependence of NP delivery on EPR is the hypothesis that NP design can facilitate endothelial transcytosis, providing an alternative pathway to the tumor [34]. NP size [34] and surface modifications with ligands for vascular and/or tumor expressing receptors [35,36] have both been investigated with promising effects on transcytosis initiation and increased nanoparticle internalization.

#### Clearance

An additional challenge to NP delivery and retention is the clearance patterns executed by the body. While clearance of NPs is an important aspect of delivery for clinical use, rapid clearance reduces NP accumulation and activity at the target site. The liver, spleen, and kidney constitute the primary organs of clearance for NPs. Recently, Tsoi *et al.* showed, through mathematical modeling, that the reduced velocity of blood flow in the liver leads to increased NP accumulation in the liver compared to tumors and other organs [37]. Avoiding rapid clearance by these organs and increasing circulation half-life within the body can be modulated by altering NP size and surface properties, as will be discussed in the next section. Buckley *et al.* tested clearance rates following the inhalation of various sized NPs from the lung, liver, and kidney, and concluded that while lung clearance showed no correlation between NP size and clearance rate, liver and kidney clearance was size-dependent [38]. This shows that NP characteristics are just one of the important factors in evaluating NP delivery and efficiency. Some polymer-NPs are coated with polyethylene glycol (PEG) to increase circulation time, which has been widely successful for synthetic and natural particles [39,40]. Hence, various design characteristics will influence the circulation of the NPs, and the design criteria should be chosen based on the disease model and application. A detailed pharmacokinetic modeling strategy is required prior to experimentation for a better understanding of the success of NPs in the desired application.

### Organ-Level Barriers

Apart from the mononuclear phagocytic system (MPS), which is a major part of the RES and poses a barrier to NP distribution, there are also barriers based on the tumor niche. To date, there is no one modification of a nanoparticle that has been able to overcome the challenges that these different biological barriers pose. While PEGylation has been shown to increase circulation time and an escape from being cleared by the MPS and RES, organ-specific architecture and resulting vascular permeability present many unique challenges to the distribution and uptake of NPs.

One illustrative example is that of the blood-brain barrier (BBB), which highly regulates the exposure of the brain to the systemic environment. Difficulties in overcoming this barrier can be seen in the static rate of poor outcomes in patients with brain cancer. Brain capillary endothelial cells (BCECs), which line the brain side of the BBB, are highly polarized, with functionally distinct luminal and abluminal membrane compartments. These cells have unique properties compared to endothelial cells found in peripheral tissues, which confer most of the selective properties of the BBB. Instead of being separated by large fenestrations, BCECs are connected by tight junctions (TJs) at the lateral, luminal membrane, which present a high-resistance barrier to the diffusion of small hydrophilic molecules and ions [41,42]. BCECs also display low rates of transcytosis, which limits the vesicle-mediated transcellular movement of solutes [43]. The tightly associated cellular components of the BBB severely restrict the passage of substances into the brain. The transport of necessary nutrients and certain drugs is regulated by a series of specific transport mechanisms. Additionally, several characteristics of a substrate affect its ability to passively diffuse across the BBB, including lipid solubility, size, polarity, concentration gradient, and the surface area for diffusion [44]. In general, small lipophilic molecules are able to passively diffuse across the BBB more easily. Muntoni *et al.* have recently shown that a new generation of lipid nanoparticles called solid lipid nanoparticles is able to cross the BBB when injected intravenously and deliver toxic drugs such as methotrexate effectively to the brain [45]. Takeuchi *et al.* have shown that PEGylation of nanoparticles can also increase accumulation in the brain when compared to non-PEGylated NPs [12]. NPs can be engineered to target many of the BBB-specific transport mechanisms in order to increase delivery efficiency while carrying an entrapped, adsorbed, or covalently bound drug. Additionally, there are various methods of temporarily disrupting the permeability of the BBB to enable NP/drug delivery, such as through the administration of hyperosmotic agents or ultrasound energy [8,46]. This was shown through the use of mannitol to disrupt BBB temporarily for delivery of drugs to the brain parenchyma [47].

The fenestrated sinusoid capillaries of bone marrow are more permissive to cancer cell infiltration or NP uptake. Studies have shown that PEG-PGLA nanoparticles, when engineered with bisphosphonate and carrying bortezomib, increase circulation (through the use of PEG/PLGA) and accumulate with higher affinity in the bone [48]. On the other hand, the design of NPs for delivery to the lung should be modified by taking into consideration the large surface area, thin alveolar epithelium, rapid absorption, lack of first-pass metabolism, high bioavailability, and the capacity to absorb large quantities of drug [49]. Recently, Zelepukin *et al.* demonstrated an approach where they used a physiological process called “RBC hitchhiking” to deliver positively charged 100 nm particles to the lung. The study demonstrated the effect of charge to be a crucial factor when testing over eight NP formulations with different surface characteristics [50]. A study using an aerosol-based formulation of albumin-encapsulated NPs also showed the accumulation of the particles with a longer retention time in the lungs. This study used the large surface area and the inherent ability of the lung parenchymal cells to uptake albumin to deliver the NP-encapsulated drug [51]. The liver, though well vascularized with a conducive environment for NP delivery and accumulation, is plagued by low clearance of NPs leading to toxicity concerns. A thorough understanding of liver-NP interaction is required for design of NPs that maintain the balance between accumulation and clearance through the organ. It is cellular heterogeneity within the liver that contributes to the fate of NPs in the liver. Kupffer cells, specialized liver resident macrophages, contribute to NP uptake, but their effects on the systemic role the liver plays in NP delivery is minimal [52]. Recently Campbell *et al.* demonstrated through the use of three different NPs that the regulation of NP accumulation and clearance in the liver is stab-2 mediated. They hypothesize that since stab-2 is not essential for normal adult physiology, targeting stab-2 could improve circulation time and decrease retention by the liver [53].

### Cellular-Level Barriers

Once at the target organ, navigating the NPs into the tumor cells poses a significant challenge. On the cellular level, the internalization of material occurs through phagocytosis, macropinocytosis, caveolin-, clathrin- or receptor-mediated endocytosis, and/or transcytosis. The pathway used by the NP is dependent on the surface characteristics of the NP, including ligand targeting and varying amounts of kinetic energy [54]. Zhang *et al.* also highlighted the significance of NP shape and size on the kinetic energy-dependent endocytic rate [55].

Two main mechanisms of cellular endocytosis found in most tumor cells are either clathrin- or caveolin-mediated. Operational endocytosis pathways vary between cell types and are influenced by changes in the extracellular environment. Understanding how the targeted cell interacts with its environment is essential to NP development since most NPs tend to aggregate or agglomerate in biological fluids leading to changes in size. Ligand conjugation via adsorption or covalent bonding allows for increased control over NP-cell interactions and can influence the process of cellular internalization, improving precise cellular targeting. Albumin and folic acid are examples of ligands which facilitate caveolin-mediated uptake of NPs [56]. The size of the NP also influences the cell intake pathway to a certain extent. Internalization of particles with a size <200 nm most often involves a clathrin-mediated mechanism, whereas with an increase in size there is a tendency towards caveolin-mediated pathways [57]. Once an engineered NP enters a cell, it also needs to be able to bypass the intracellular endocytic pathway leading to the lysosome. Clathrin-mediated endocytosis eventually leads to degradation in the lysosome whereas the caveolin-mediated pathway does not. Therefore, in the case of clathrin-mediated uptake, endosomal escape of NPs must occur prior to lysosome degradation [56]. Using poly(ethyleneimine) (PEI), Galliani *et al.* were able to show successful endosomal escape and cytoplasmic delivery of their nanoparticle cargo [58]. Actively targeting NPs to tumor-overexpressed receptors may also enable receptor-mediated cellular internalization without degradation by lysosomal compartments.

## NANOPARTICLE DESIGN CHARACTERISTICS TO OVERCOME BARRIERS

The most common and practical approach for successful and efficient delivery has been to target the various characteristics of nanoparticles that can influence the interactions with these biological barriers. Nanoparticle material, size, and surface characteristics greatly influence the ability of NPs to effectively reach and interact with target organs and cells.

### Material-Based Modifications

#### Liposomes

Liposomes are spherical particles in a colloidal dispersion which are composed of phospholipid molecules that form enclosed lipid bilayers [59]. The assembly of a liposome is rather straightforward, as the amphiphilic nature of a phospholipid and the thermodynamic properties of the aqueous environment drive self-assembly into an entropically favorable orientation with a hydrophobic segment enclosed within the nanoparticle core [60]. The amphiphilic phospholipid bilayer of liposomes has close resemblance to the mammalian cell membrane, enabling efficient interactions between liposomes and cell membranes and subsequently effective cellular uptake. Liposomes tend to have short circulation times, and this has been overcome by adding PEG to generate “stealth liposomes” that can escape opsonization and prolong circulation times [61–63]. Targeted liposomes, such as glutathione-conjugated liposomes and heat-responsive liposomes, have been shown to cross the BBB for increased tumor penetration and accumulation [64,65]. Success has also been seen with a multivesicular liposomal platform, called the DepoFoam technology, for sustained or extended release of encapsulated drugs that require multiple dosings over time. Depocyt, administered through spinal injections, was approved in 1999 for neoplastic meningitis [66].

#### Protein-encapsulated nanocarriers

Various types of proteins ranging from animal-based protein such as albumin, collagen, and gelatin, to plant-based protein, such as ferritin, have been investigated for their use in nanomedicine [67,68]. Animal protein-based nanoparticles possess outstanding biodegradability along with low toxicity of by- and end-products. Specifically, the use of albumin-based nanoparticles for biomedical applications has been researched since 1972 [67,69,70]. The first protein-based nanoparticle to be approved by the FDA is Abraxane®, an albumin-bound paclitaxel nanoparticle formulation used in combination with chemotherapeutics for the treatment of several cancers. Of note, a combination therapy with gemcitabine and Abraxane® has shown improved results in patients with orphan disease pancreatic metastatic cancer [18]. This NP formulation improved the bioavailability of paclitaxel, a chemotherapy medication, with four times longer half-life, 43% slower clearance, and increased local concentration of therapeutics within the tumor mass [18,71]. As an endogenous component of human blood, albumin shows no immunogenicity upon administration. The multiple internal binding pockets and an external free thiol group provide albumin with the versatility to bind to multiple drug formulations [72], which has led to additional albumin-drug NP combinations including curcumin and doxorubicin [73], cabazitaxel [74], and others. Albumin NPs also present a long half-life, which maintains high drug concentration in circulation [39]. Recently, a number of additional formulations with albumin encapsulation have been studied, including a formulation of human albumin fragments as nanocarriers of paclitaxel to improve antitumor efficacy through improved release of the drug [75]. With many advantages of protein-based NPs to facilitate their clinical applications and the success of Abraxane®, these NPs have great potential in other drug delivery areas, such as bio-imaging and as theranostic agents.

#### Polymeric nanoparticles

Polymeric nanoparticles are organic polymer compound assemblies in the form of nanospheres (solid spheres) or nanocapsules (hollow spheres with a void space in the center). The compact assembly of the outer particle layer in polymeric nanocapsules enables better drug retention, leading to enhanced delivery to the disease site [17,76]. The characteristics of the polymer, such as charge, functional group variation, and length of the main carbon chain, can also be easily manipulated. These features allow the NP to achieve high biodegradability, long circulation time, the ability to target specific disease locations of interest, and controlled release [77]. This was demonstrated in a study where proteasome inhibitors (such as MG132) administered by themselves show poor selectivity and specificity owing to the exposure of the aldehyde bond. However, when administered in a polymeric micelle, which shielded the aldehyde bond, the therapeutic function of MG132 was restored [78]. Polymeric NPs, a variety of which have been designed and applied in both preclinical and clinical studies, offer several additional advantages, such as a controlled release profile from the structure matrix, encapsulation of labile molecules (DNA, RNA, and proteins), and excellent *in vivo* stability [79]. These features illustrate polymeric NPs’ potential in drug delivery, imaging, and theranostic applications.

#### Inorganic nanoparticles

*Metallic nanoparticles* are a major class of inorganic nanoparticles, commonly developed with a metal element and their oxide derivatives, such as gold, silver and aluminum oxide, cobalt oxide, iron oxide, titanium dioxide, and zinc oxide [80,81]. The magnetic properties of these metallic NPs could be used for magnetic guidance during therapy as well as for hypothermic treatment via a magnetic field-induced temperature increase [82]. Moreover, the high density of free electrons in the valence band in these metal ions, results in an interaction between these free electrons and the excitation phase, making them excellent contrast agents for imaging purposes, such as enhanced magnetic resonance imaging (MRI) [83]. Nanotherm®, a tumor therapy drug consisting of aminosilane-coated superparamagnetic iron oxide nanoparticles (SPION), was approved in Europe in 2010 for its use in glioblastoma therapy based on magnetic hyperthermia. It has been shown to increase overall patient survival by up to 12 months [18]. The incorporation of hybrid metallic materials into one nano-entity could enable the use of multimodal agents for imaging purposes [84,85]. Near-infrared (NIR)-based gold and MRI-functional iron oxide has been a popular hybrid NP used in MRI. Research has shown that by integrating gold and copper ions with organic dyes into a nanoporphyrin structure, it can enable access to multiple imaging and therapeutic platforms, including NIR fluorescence imaging, MRI, positron-emission tomography (PET), photothermal, and photodynamic therapies [84]. Metal NPs therefore have potential in both therapy and diagnosis due to their unique magnetic responsive properties, although further research into the toxicity profile and induced immune response has to be performed to justify clinical application.

*Quantum dots (QDs)* are small nanocrystals (2–10 nm) made of semiconducting material and were first discovered in 1980. They can be lead or cadmium based with emission spectra that can be tuned based on their core-shell structure, size, and density states [86,87]. One of the strengths of QDs is their emission in the near-infrared (NIR) region which is promising for *in vivo* work where tissue absorbance and light scattering interferes significantly with imaging tools [87]. QDs, while highly stable as imaging agents, are cytotoxic in nature [88], limiting their use in tumor imaging.

*Rare-Earth based NPs,* a new class of optical nanoprobes, utilize shortwave-infrared (SWIR) light emission (1000–3000 nm) from rare-earth (RE) doped phosphors [89]. A unique property of the RE SWIR probes is their ability to emit detectable luminescence from depths beyond those possible with NIR or visible modalities in biological tissues [89]. Two additional advantages of these probes are (1) compatibility with low power excitation sources, and (2) characteristic optical emissions within narrow spectral bandwidths (<50 nm) for multiplexing. The excitation and emission wavelengths of these probes can be tuned by modifying the RE core dopant(s) and host matrix chemistry. These probes have been encapsulated into rare earth albumin nanocomposites (ReANCs) and used as imaging tracers and theranostic particles for cancer targeting, tumor penetration, and drug delivery [89–91].

### Size-Based Modifications

**Figure 2.**
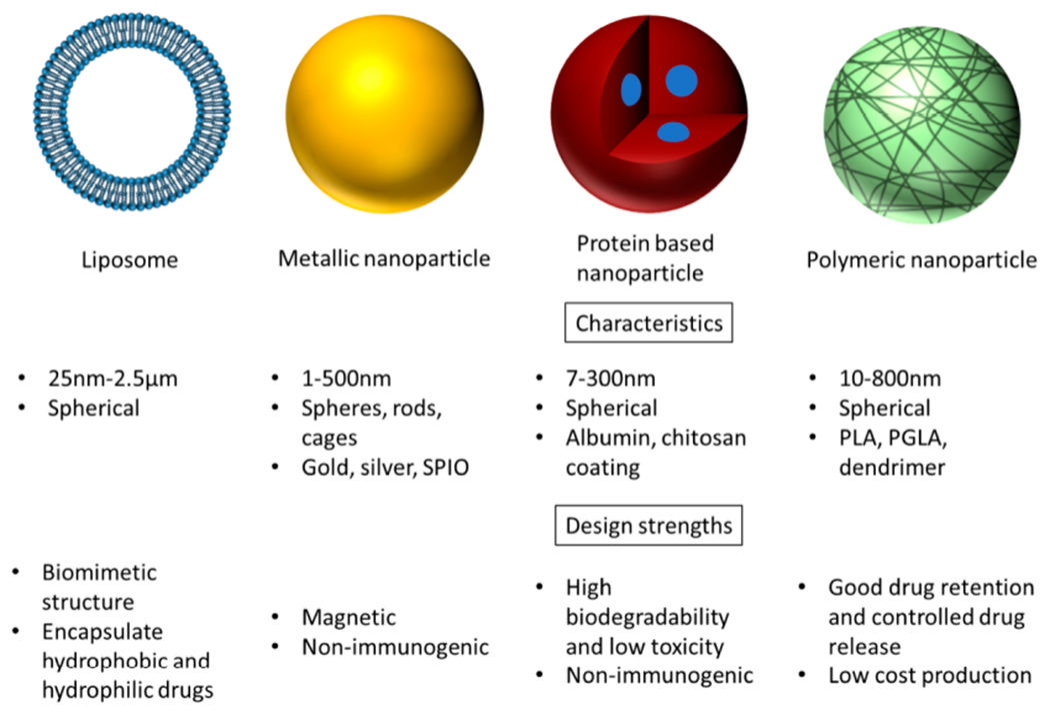
Nanoparticle classification and design characteristics.

The characteristics of nanoparticles can be engineered to overcome the limitations for each biological barrier based on size, charge, and surface modifications (Figure 2).

#### Size

Size plays a major role in determining *in vivo* biodistribution and clearance of NPs. These effects have been extensively studied in the case of the spherical geometry of NPs. The ideal range for cancer applications is estimated to be within the range of ~10–200 nm. For overall ideal distribution of a drug, nanocarriers should have good circulation half-life and efficient clearance rates to avoid toxicity by prolonged retention. The endothelial cell layer present on the interior surface of every blood vessel and lymphatic vessel forms a dynamic interface involved in the transport of essential factors and macromolecules. Although the gap between endothelial cells depends on the organ and the specific tissue environment, the average pore size of a typical endothelial layer is 5 nm [92]. Hence, particles <5 nm are excreted with very limited circulation half-life and are rapidly cleared via extravasation and/or renal clearance [92]. With an increase in size (>5 nm), an increase in circulation half-life is observed owing to reduced filtration by the glomerular capillaries combined with slower transportation across the endothelial layer. Particles with size ~10 nm tend to show prolonged circulation, with the kidneys being the primary organ of clearance [93]. The RES, specifically in the liver and spleen, becomes the primary source of clearance for particles in the range of 20–100 nm.

The size of NPs also facilitates organ-specific movement. Metallic NPs <10 nm have been shown to cross the BBB in a size-dependent manner, allowing for potential NP drug delivery to brain cancers. Gold NPs have been shown to cross the BBB and accumulate in the brain via passive diffusion through ion channels; silver and titanium dioxide NPs migrate into the brain by decreasing and disrupting the tight junctions between BCECs [94,95]. The NP material influences the size and the flexibility which can be optimized to improve site-specific delivery. The bending stiffness of the liposome membrane plays a key role in liposome encapsulation and thus affects vesicle sizes. It has been shown that higher saturated fat concentration, such as cholesterol, results in an increased vesicle peak size, distribution width, and membrane thickness [96]. Particle size can be controlled conveniently by varying metal salt solution concentrations during fabrication or by limiting the usage of strong reducing agents such as hydrazine [97]. The size and shape of QDs can be controlled with the desired packing geometries or modified with focused ion or laser beams [86]. The sizes of ReANCs are tunable and can be controlled by adjusting the pH and the salt concentration of the albumin solution prior to encapsulation [98].

#### Charge-based modifications

Charge is a key determinant of cellular localization, where highly positively charged NPs tend to show higher cellular uptake compared to neutral or negatively charged particles. However, this high rate of accumulation also leads to increased non-specific binding to normal cells, and to cytotoxicity combined with a short half-life. In contrast, negatively charged NPs have very limited uptake in cells [99]. The walls of blood vessels are negatively charged which may cause repulsion to high negative charge-bearing particles. In addition to having an effect on cellular localization, NP surface charge can also vary the overall biodistribution. For example, positively charged particles show enhanced penetration of the otherwise protected BBB [100], which overshadows the need to reduce non-specific interactions. Several types of cationic NPs have been reported to cross the BBB via the mechanism of adsorptive-mediated transcytosis (AMT), interacting with the negatively charged surface of the BCECs.

In this era of gene therapy, cationic NPs have gained more interest in cancer research in the past decade [6,101,102]. The most common method of conferring a positive charge on the surface of NPs is by fabricating NPs from multiple components that carry a positive charge at physiological pH. Cationic nanovesicles can be prepared by self-assembling bolaamphiphiles (molecules containing two hydrophilic head groups at each end of a hydrophobic chain) and used to deliver encapsulated materials into the brain [103]. Cationic NPs have also been synthesized entirely using cationic polymers such as chitosan or PEI and successfully used for brain delivery [104,105] and other solid tumor transport [106,107]. NPs can easily be formed between these positively charged polymers and negatively charged nucleic acids via formation of polyelectrolyte complexes or controlled coacervation, making them well suited for nucleic acid delivery [108,109]. The surface of NPs can be functionalized with positively charged biomolecules such as cationic albumin (functionally modified with cationic groups), PEI, or cell-penetrating cationic peptides such as TAT (transduction domain of human immunodeficiency virus type-1 (HIV-1)) peptides [110–112]. Although engineering NPs with a positively charged surface can improve transport across the BBB, these cationized NPs can have an immediate toxic effect which includes a general increase in BBB permeability [113]. The key is to find a balance between effective cellular interaction and minimal non-specific binding and toxicity. To this end, particles engineered with low negative or low positive charge have been shown to be optimal for most common applications.

## TUMOR-TARGETING NANOPARTICLES

Nanoparticle design needs to take into consideration these barriers of infiltration for effective delivery and accumulation (Figure 1). Passive targeting to tumors has been generally possible via the EPR as discussed previously and by PEGylated delivery systems, however the results for optimal pharmacokinetic values are often varied [114]. Active targeting by virtue of high ligand density on nanocarriers offers improved pharmacokinetic properties. The FDA has approved a number of nanoscale delivery systems for use in cancer and the list includes polymeric NPs, lipid-based carriers such as liposomes and micelles, dendrimers, carbon nanotubes, and gold nanoparticles [115–117]. These nanosystems have been used for drug delivery, imaging, and photothermal ablation of tumors to name a few [118]. This is an emerging field with a handful of targeted nanocarrier systems in clinical trials including those nanoparticles listed below (Table 1) [119].

The development of active targeting is a design strategy employed in current NP development to overcome barriers at a cellular level and ensure higher specificity in delivery to cancer cells. The common mode of active targeting utilizes receptor-mediated uptake, thereby increasing cellular affinity for NPs [120]. The specificity leading to reduced off-target interactions with healthy tissue is highly beneficial in therapeutics and contrast agents, in delivering effective dosages, and reducing toxic side effects [121–124]. While targeting the well-established biomarkers (receptors in most instances) using their respective ligands (small molecules, peptides, carbohydrates, antibodies, and aptamers) is an attractive option, although the binding site barrier (BSB), which was initially observed in the non-uniform binding of antibodies to tumors [125], often limits the efficiency of NPs. Miao *et al.* recently elucidated the role of the BSB, as elicited by tumor-associated fibroblasts in the stroma, in NP targeting and accumulation in tumors [126]. They showed that the expression of the targeted receptor on tumor-associated fibroblasts served as a barrier for tumor accumulation of these NPs. Through mathematical modeling it was established that the accumulation of the targeted NPs was a function of increased binding and uptake by the fibroblast layer, thus reducing diffusivity into the tumor. This could actually reduce the efficacy of the drug since fibroblasts are resistant to the effects of most anti-tumor drugs. Hence, the BSB should be a key factor while considering the design criteria for active targeting.

### Tumor-Niche Specific Nanoparticle Design

Taking cues from the nature of biological molecules and their transit in the three liters of plasma in circulation, NPs can be designed to target various tumor niches, such as the tumor microenvironment or specific tumors based on their organ of origin. For instance, to keep NPs in circulation longer they could be modified using commonly circulating proteins such as albumin, or the shape could be modified to mimic red blood cells (RBCs) to allow for improved flow and adhesion characteristics in circulation. Research has shown that RBCs could be used as carriers of nanoparticles themselves to avoid immune-escape and prolong circulation time, as recently reviewed by Xia *et al.* [124]. Also, in circulation as well as in the tumor microenvironment, exosomes and other endogenous vesicles successfully interact with tumor cells, the understanding of which can be applied to improve NP design. As an endogenous material, exosomes without modifications do not carry the same risk of toxicity as engineered NPs, can cross natural intracellular and extracellular barriers [127,128], and participate in targeted uptake [129]. These properties are key to enhancing NP delivery and if used as a template, may improve NP design and delivery success. Furthermore, merging SPIONs and exosomes has shown enhanced cancer cell targeting and killing [130] suggesting the possibility of not only using the two vesicle types as models for the other, but combining both to take advantage of their respective strengths.

Tumors themselves present unique environments that influence NP accumulation and retention. Here we present an example each of two tissue architectures, one that is highly contiguous (lung) and does not permit passive infiltration of NPs and the other being fenestrated and discontiguous (bone) allowing for easier NP infiltration.

### Targeting to Lung Lesions

A number of NP formulations such as aerosols [131,132] and liposomes [133,134] are being currently evaluated and will need to balance the optimal threshold of drug loading without causing therapy resistance while assessing safety of prolonged circulation time in the lung microenvironment. The three goals of targeting lung lesions using NPs are: (1) lung retention; (2) prevention of rapid clearance by lung macrophages; and (3) initiation of multifunctional therapeutic payload delivery. The challenges facing the clinical use of NPs for lung targeting include non-uniform size distribution of NPs, toxicity, and reproducibility [135]. Delivery of NPs as aerosols has gained momentum in the treatment of lung cancer since this can also reduce the total drug dose required and improve accumulation, improved bioavailability, and retention of the drug at the target site [136,137]. NPs can be modified by surface modifications such as coating with PEI, chitosan, *etc.* to prevent macrophage clearance and improve residence time in the lungs [138]. A number of inhalation therapeutics for lung cancer is being explored in the form of polymeric NPs [139,140]; solid lipid NPs [141–143]; and polymer-drug conjugates [144], as well as others. A key consideration while working with inhalation- or aerosol-based NPs is toxicity and future studies will be required to assess optimal particle characteristics, systemic/non-pulmonary biodistribution and their effect on other vital organs.

### Targeting to Bone Lesions

As described earlier, the discontiguous fenestrated architecture of the bone allows for a more flexible NP design since the barrier to infiltration is reduced, allowing for prolonged circulation time. NP design in such a niche can then focus on increased therapeutic loads by attaching the therapeutic to the carrier via a linkage susceptible to proteases such as matrix metalloproteinases (MMPs) and prolonged residence time in the bone as shown recently by Ross *et al.*, where a nanocarrier with docetaxel payload targeting the integrin β3 for delivery to bone showed superior efficacy in a breast cancer bone metastasis model [145].

## APPLICATIONS OF NANOPARTICLES

Nanomaterials provide targeted delivery of therapeutics to disease sites, as well as acting as contrast agents themselves or as delivery vehicles for exogenous contrast agents. NPs possess better targeting capabilities, increasing the signal to noise ratio compared to conventional imaging agents.

### Therapeutics

For nanomaterial-based therapeutics, liposomes have been the most successful formulation for clinical application to date, as seen with Doxil® [16]. The liposomal formulation of this anticancer drug not only provides a long half-life and enhanced tumor deposition, but also lowers the incidence of cardiotoxicity, myelosuppression, alopecia, and nausea [7,17]. NP formulations using liposome-derived NPs for the treatment and diagnosis of breast cancer have also shown promise in improving drug efficacy with targeted delivery and prolonged circulation in the system and reducing side-effect on other organs caused by chemotherapy drugs [146]. Abraxane® has been approved for the use of breast cancer treatment, while three other NP formulations, including liposomal Paclitaxel, liposomal Cisplatin, and PEGylated liposomal Irinotecan, are going through clinical trial approval [147]. Recent research has shown 100 nm PEGylated liposomes to target triple negative murine breast cancer metastasis and suggest the possibility of targeting the pre-metastatic niche to prevent further metastatic progression [148]. The challenge of glioblastoma therapy lies in the genetic and signaling heterogeneity and the ineffective delivery method hindered by the presence of the BBB, both of which make therapy insufficient to reverse tumor progression [149]. In the past decade some hope has emerged with the development of lipopolymeric NPs that enable efficient delivery of therapeutics (such as RNAi) into tumor cell matrix and nanoencapsulated siRNA has been shown to be effective at suppressing tumor growth [150]. The success of liposomes in the clinical arena is based on the flexibility of the material. Besides their structural similarity to mammalian cell membranes, another key feature of liposomes is that their phospholipid bilayer structure enables the encapsulation of both hydrophobic drugs, which have high affinity to the bilayer (e.g., Ambisome®, trapped amphotericin B), and hydrophilic drugs, which are encapsulated inside the aqueous core (e.g., Doxil®, encapsulated doxorubicin) [151,152]. Liposomes’ enhanced drug delivery to disease locations and their promotion of specific cell targeting within the disease site have achieved clinical acceptance and established their position in modern drug delivery systems.

### Diagnostics

In addition to using these particles as nanocarriers of drugs, imaging and diagnostic platforms are being revolutionized by the application of nanomaterials. In the place of drugs or therapeutics, NPs can be loaded with imaging dyes or other materials. However, the major advantages of these materials over existing non-NP based contrast agents is that they increase imaging material half-life [153] and can be modified for (1) targeted/precision detection by molecular targeting through surface modification with tumor-specific biomarkers, thus serving to molecularly phenotype tumors, and (2) they can be designed to carry multiple payloads to serve as theranostics (see section below). For example, targeted nanoformulations with gadolinium for MRI contrast [154] as shown by Zhao *et al.* using transferrin as a targeting agent [155] are emerging. Additionally, many NP formulations provide innate properties that facilitate imaging and diagnostic capabilities, without the need for exogenous cargo.

The use of near-infrared (NIR) dyes such as QDs reduces tissue absorption when compared to dyes that emit in the visible range, however the challenge of deep tissue penetration needs to be overcome with QDs. Recently, rare-earth nanomaterials (REs) that are bright, stable, tunable, and emit in the short wave infrared (SWIR) region, which overlaps with the “second and third optical windows” from 1000 to 1600 nm, have been developed to overcome the issues of tissue absorption, interference from autofluorescence [156], and deep tissue penetration. These offer superior detection sensitivity and the capability of multispectral *in vivo* SWIR imaging. ReANCs have been used to detect emerging and disseminated tumors in melanoma, breast and ovarian cancer mouse models [89–91,157–159].

Another study showed SPIONs’ ability to provide contrast-enhanced MRI of primary breast tumors *in vivo* and thus have the potential for MRI detection of micro-metastases, suggesting that metallic NPs with unique magnetic properties are a promising platform for future breast cancer therapy [160]. Ferumoxytol®, iron oxide NPs coated with polyglucose sorbitol carboxymethyl ether, have been broadly investigated for clinical imaging of various cancer pathologies and photothermal tumor ablation in preclinical settings [161,162]. Wei *et al.* have developed zwitterion-coated SPIONs that have shown pre-clinical success as an MRI contrast agent in comparison to the gadolinium gold standard [163]. Additionally, the potential dual advantage of using NPs for MRI contrast and therapeutic shuttles has been shown by Luo et al, with successful knockdown of PD-L1 with a SPION/siRNA complex [164].

### Theranostics

NPs offer the unique advantage of modifications that can increase the number of modalities to be used with one dose or injection. These have taken on the term nanotheranostics, illustrating their potential for both therapy and diagnosis. Nanotheranostics such as molecularly targeted QDs [87] and ReANCs [90,91,157] provide the promise of detection and treatment in a precise manner.

Molecularly tailored imaging techniques [165] paired with a therapeutic will provide more information on pharmacokinetics at the lesion site and on the unwanted side effects occurring due to accumulation in off-target sites. Design parameters should take advantage of the surrounding environment, such as the increase in proteases in the tumor microenvironment, and design drugs to be covalently linked using protease cleavable linkers. The major challenge that will remain in the successful development of nanotheranostics will be the balance between targeting and drug dosing, as well as maintaining ligand density for sufficient accumulation to ensure increased signal from target sites while loading the appropriate drug dose that will yield a high therapeutic index for desired pharmacological effects.

## CONCLUSION

The key challenge in nanoengineering will be the design of a formulation that will provide both specificity and potency. The gap between NP design and understanding nano-bio interactions needs to be bridged for successful next-generation precision nanoengineered platforms that can not only deliver drugs but also illuminate the site of delivery for a non-invasive real-time monitoring approach.

## Figures and Tables

**Table 1.** Nanoparticles with FDA approval or currently in at least a Phase III clinical trial for cancer therapy and diagnostics (http://www.ClinicalTrials.gov) [18].

Nanoparticle name	NP formulation	Cancer targets	Trial name, status
**Doxil®, Myocet®, Caelyx®**	Doxorubicin-loaded liposome	Ovarian, Kaposi’s sarcoma, multiple myeloma, breast	FDA approval 1995
**DaunoXome®**	Liposomal daunorubicin	Kaposi’s sarcoma	FDA approval 1996
**Abraxane®, ABI-007**	Albumin-bound paclitaxel	Breast, lung, pancreatic cancer, melanoma	FDA approval 2005
**Nanotherm®**	Iron oxide nanoparticle	Glioblastoma	EU approval 2010
**Marqibo®**	Liposome vincristine	Acute lymphoblastic leukemia	FDA approval 2012
**Onivyde®**	Irinotecan-loaded liposome	Metastatic pancreatic cancer	FDA approval 2015
**Vyxeos®**	Daunorubicin and cytarabine loaded liposome	Acute myeloid leukemia	FDA approval 2017
**SPIO MRI/Ferumoxytol®**	Superparamagnetic iron oxide nanoparticles + MRI	Pancreatic cancer metastasis	Phase IV (2008–2017)
**NK105**	Paclitaxel-containing polymeric micelle	Breast cancer recurrence	Phase III (2015–2020)
**NBTXR3**	Crystalline NP + radiation	Soft tissue sarcoma	Phase II//III (2015–2020)

MRI, magnetic resonance imaging.
